# Can biochemical pregnancy be determined 5 days after frozen-thawed embryo transfer?

**DOI:** 10.5935/1518-0557.20210054

**Published:** 2022

**Authors:** Isaac M Yadid, Thelma S Criscuolo, Jéssica F Santos, Luiz A Giordano

**Affiliations:** 1 Clínica Primordia Medicina Reprodutiva, Rio de Janeiro, RJ, Brazil; 2 Instituto de Biofísica Carlos Chagas Filho, Universidade Federal do Rio de Janeiro (UFRJ), Rio de Janeiro, RJ, Brazil; 3 Departamento de Biologia Geral, Laboratório de Genética Humana e Mutagênese - Universidade Federal da Bahia (UFBA), Salvador, BA, Brazil; 4 Hospital Universitário Gaffrée e Guinle, Universidade Federal do Estado do Rio de Janeiro (UNIRIO), Rio de Janeiro, RJ, Brazil

**Keywords:** β-hCG, biochemical pregnancy test, frozen-thawed embryo transfer

## Abstract

**Objective:**

To study the predictive value of serum β-hCG on day 5 after frozen-thawed embryo transfer (FET) to predict pregnancy outcomes and to establish cut-off values for very early biochemical pregnancy diagnosis.

**Methods:**

This retrospective cohort study was performed at a private reproductive medicine centre and we reviewed the medical records of women who underwent FET cycles from January 2018 to June 2019. A total of 116 evaluated cycles had serum β-hCG levels measured on days 5 and 10 after FET. The predictive value of serum β-hCG levels measured on day 5 after FET was investigated for very early biochemical pregnancy diagnosis.

**Results:**

The standard biochemical pregnancy diagnosis was defined as a β-hCG ≥25 IU/L on day 10 after FET. We then generated a receiver operating characteristic curve, and the cut-off value of β-hCG on day 5 for predicting biochemical pregnancies was 4.0 IU/L, with 93.4% sensitivity and 92.7% specificity (AUC, 0.960; 95% confidence interval, 0.923-0.997).

**Conclusions:**

Values for β-hCG at day 5 after FET ≥4.0IU/L are accurate for the diagnosis of biochemical pregnancy. The use of very early biochemical pregnancy diagnosis in clinical practice enables earlier management, patient counselling, and appropriate follow-up.

## INTRODUCTION

Human chorionic gonadotropin (hCG) is the first hormonal signal of the presence of an embryo. Blastocysts express hCG even before implantation, and its production by the syncytiotrophoblast increases further after implantation, until peak hCG production between the 10^th^ and 11^th^ week of gestation (Makrigiannakis *et al*., 2017). The detection of hCG has been used to detect pregnancy since the 1920s. Knowledge concerning hCG physiology and the evolution of bioassays enabled the introduction of radioimmunoassay and commercial hCG tests at the end of the 1960s. In 1973, a radioimmunoassay that specifically detected the hCG β subunit (β-hCG) was introduced and led to more reliable pregnancy tests. Immunoassays can detect hCG tracers through sensitive antibody enzyme labelling and high sensitivity fluorometric and chemiluminescent methods (Cole, 2009). β-hCG levels can be measured in the maternal blood as early as 10 days after fertilization, and pregnancy can be detected soon after missing menses (Makrigiannakis *et al*., 2017).

In *in vitro* fertilization (IVF) cycles, embryos are usually transferred to the uterus on days 3 to 5 of development, and a single determination of maternal serum β-hCG concentration 11-12 days after embryo transfer is a reliable indicator of pregnancy that is generally used by reproductive medicine specialists (Bjercke *et al*., 1999). A number of studies have shown that the β-hCG levels after IVF cycles can be used to predict a viable pregnancy and the results can be interpreted using different cut-off values depending on the situation (Heiner *et al*., 1992; Schmidt *et al*., 1994; Fridström *et al*., 1995; Glatstein *et al.*, 1995; Guth *et al*., 1995; Qasim *et al*., 1996; Chen *et al*., 1997; Bjercke *et al*., 1999; Homan *et al.*, 2000; Sugantha *et al*., 2000; Papageorgiou *et al*., 2001; Poikkeus *et al.*, 2002; Urbancsek *et al*., 2002; Carmona *et al*., 2003; Lambers *et al*., 2006; Stone *et al*., 2006; Porat *et al.*, 2007; Delbaere *et al.*, 2008; Shamonki *et al*., 2009; Basirat & Bijani, 2010; Chi *et al*., 2010; Kathiresan *et al*., 2011; Lawler *et al*., 2011; Reljič *et al*., 2013; Asvold *et al.*, 2014; Sung *et al*., 2016; Dypvik *et al.*, 2018).

Under physiological conditions, a positive pregnancy is generally indicated when β-hCG levels reach 25 mIU/ml 10 days after conception and increase exponentially thereafter, doubling roughly every 2-3 days for the first 4 weeks of pregnancy. In contrast, levels below 5 mIU/mL exclude pregnancy, and slower than expected increases in β-hCG indicate abnormal outcomes (Fan *et al*., 2017). This pattern is similar in both natural and IVF conceptions. Therefore, β-hCG levels are traditionally measured after missing menses in natural conceptions or from 10 days after embryo transfer in IVF cycles, which correspond to around 15 days after conception in both situations (Lenton *et al.*, 1991). The urine test can be positive about 12 to 14 days after conception. Thus, once the patients go through the urine test, we believe that the greater sensitivity of the blood test would be capable of an earlier diagnosis.

There is no study showing the β-hCG measurement before 10 days after embryo transfer in IVF cycles for biochemical pregnancy diagnosis. However, in clinical practice there are a number of patients who run urine pregnancy tests sold over-the-counter in order to obtain earlier answer about the success of their treatment and obtain positive results. The high sensitivity of commercial tests available today, especially serum measurements, enables serum β-hCG detection levels as low as 0.1 mIU/mL. Based on this, we hypothesized that serum β-hCG levels could be measured as soon as 5 days after embryo transfer in order to predict pregnancy following IVF cycles. Thus, the aim of this study was to evaluate the feasibility of serum β-hCG measurement on day 5 after embryo transfer to predict pregnancy outcomes in frozen-thawed IVF cycles, and establish cut-off values for that.

## METHODS

### Study population

This was a retrospective cohort study that included the medical records of women who underwent frozen-thawed embryo transfer (FET) cycles from January 2018 to June 2019 at a private reproductive medicine center in Brazil. All procedures performed in the patients included were part of the routine care in our center, and we obtained written informed consent from all patients before treatment. The patients consented to the diagnostic and treatment procedures, and to the use of retrospective data for scientific publications, respecting anonymity. Consequently, the study was exempt from approval by the Institutional Review Board.

We reviewed the medical records of the patients, and inclusion criteria were FET cycles and determination of serum β-hCG measurements on days 5 and 10 after embryo transfers. Among 698 eligible FET cycles, 132 patients had determination of serum β-hCG on days 5 and 10. From those we excluded 16 cycles in which the embryos were biopsied for preimplantation genetic testing for aneuploidies. As a result, the study samples comprised 116 FET cycles.

### Treatment protocol

All the patients underwent a short protocol for ovarian stimulation according to routine and standard protocols. Briefly, pituitary blockage was performed with a gonadotropin-releasing hormone (GnRH) antagonist, and we achieved ovarian stimulation using recombinant FSH associated or not to recombinant LH or human menopausal gonadotropin (HMG), with flexible dose adjustments as necessary. When at least two leading follicles reached 18 mm, final oocyte maturation was triggered by a GnRH agonist or hCG, and oocyte pickup was performed 36-38 hours later. We fertilized the oocytes through intracytoplasmic sperm injection, and evaluated the embryos by morphological criteria; blastocysts were considered of top quality when they were expanded (grade 4 or 5), and both the inner cell mass and the trophectoderm were classified as A (Gardner *et al*., 2000). Viable embryos were either transferred or cryopreserved for further frozen-thawed embryo transfer.

For the frozen-thawed embryo transfers, hormone replacement cycles were accomplished with oral estradiol valerate (Primogyna, 2 mg every 8 hours) or transdermal gel (Oestrogel, 2 pumps every 12 hours), starting on the second or third day of the menstrual cycle. When the endometrium reached at least 7 mm, micronized progesterone was administered orally (Utrogestan, 200 mg every 12 hours), and vaginal micronized progesterone (Crinone 8%, one application a day) was added until at least the point of the pregnancy test. For the frozen-thawed embryo transfers, embryos were warmed, evaluated for survival and morphology, and transferred at day 5 or 6 of development. One to three blastocysts were transferred according to the patient age following the Brazilian recommendation (CFM, 2017).

### Serum hormone measurements

The β-hCG Serum concentrations were measured on days 5 and 10 after blastocyst transfers in the peripheral blood at tertiary laboratories and results were expressed in IU/L. Women were oriented to collect blood on day 5 afterward at least 120 hours after FET for β-hCG detection. We collected the data from medical records, and we used the β-hCG on day 10 after blastocyst transfer as the gold standard for pregnancy diagnosis, in which values ≥25IU/L were considered positive biochemical pregnancies. We also measured the serum estradiol (E2) and progesterone (P4) concentrations.

### Data analysis

All statistical analyses were conducted using the IBM SPSS Statistics Version 21.0 (IBM Corp., USA). Means and standard deviations were calculated for continuous variables, and Student's t-test was used for comparisons. We used the Chi-square test or the Fisher's exact test to compare the frequencies and proportions, as appropriate. Receiver operator characteristic (ROC) curves were constructed to determine the sensitivity and specificity for biochemical pregnancy prediction based on the β-hCG levels on day 5 after blastocyst transfer, and to determine the cut-off value that best discriminated between positive and negative biochemical pregnancies. Statistical significance was set at *p*≤0.05.

## RESULTS

The included patients were 37.8±4.4 years old. There was a single embryo transfer for 32 women (27.6%), two embryos were transferred for 78 women (67.2%), and six patients had three embryos transferred (5.2%). The biochemical pregnancy rate based on the β-hCG levels on day 10 was 52.6%. Table 1 demonstrates the general characteristics of the total study population, and those with negative and positive biochemical pregnancy according to β-hCG levels on day 10.

**Table 1.** Patient characteristics, cycle parameters, and serum β-hCG levels (mean ± SD).

	Total ofpatients(mean ± SD)	Negativebiochemicalpregnancy(mean ± SD)	Positivebiochemicalpregnancy(mean ± SD)	*p* *
N	116	55	61
Age (years)	37.8±4.4	37.6±4.6	38.0±4.2	0.656
No. of embryos transferred	1.8±0.5	1.7±0.5	1.9±0.5	0.050
β-hCG day 5 (IU/L)	11.8±15.4	1.6±2.9	21.0±16.3	< 0.001
E2 day 5 (pg/mL)	198.6±109.1	180.9±93.5	213.5±119.4	0.109
P4 day 5 (ng/mL)	56.0±100.5	60.8±109.6	51.8±92.6	0.639
β-hCG day 10 (IU/L)	174.3±228.3	1.7±4.0	330.0±218.9	< 0.001
E2 day 10 (pg/mL)	224.8±161.8	175.2±101.9	266.3 ±189.7	0.004
P4 day 10 (ng/mL)	53.7±107.3	52.8±107.9	54.5±107.8	0.939

Total patient data were divided into two groups according to biochemical pregnancy outcome (β-hCG levels on day 10 ≥ 25 IU/L).

* Student t-test comparing means between subgroups of negative and positive biochemical pregnancy based on β-hCG levels on day 10.

**Figure 1:**
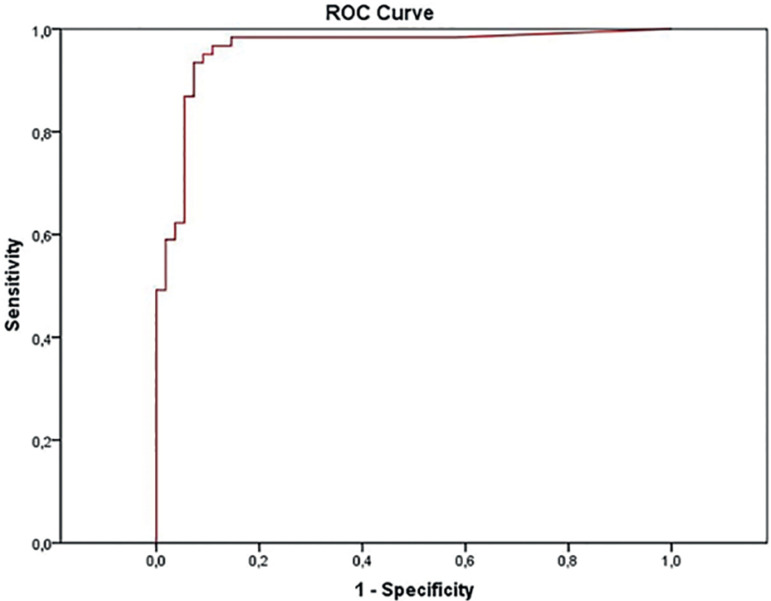
Receiver operating characteristic curve for predicting biochemical pregnancy from serum β-hCG concentration on day 5 after blastocyst transfer in frozenthawed cycles. The y-axis represents the sensitivity and the x-axis 1-especificity.

Among the 61 patients who had positive β-hCG levels on day 10 (≥ 25 IU/L), 60 also had positive β-hCG levels on day 5, varying from 2.2 to 75.5 IU/L. Considering β-hCG levels on day 10 ≥ 25 IU/L as standard, a ROC curve analysis showed that 4.0 IU/L is the cut-off value of β-hCG on day 5 for biochemical pregnancy diagnosis. That analysis had 93.4% sensitivity and 92.7% specificity (AUC, 0.960; 95% confidence interval, 0.923-0.997) (Figure 1).

If we consider the β-hCG concentration on day of 5 ≥ 4.0 IU/L to diagnose biochemical pregnancy, the concordance between positive tests based on days 5 (β-hCG ≥ 4.0 IU/L) and 10 (β-hCG ≥ 25.0 IU/L) was high (measure of agreement Kappa, 0.86; *p*<0.001). Moreover, the positive predictive value (PPV) was 93.4%, and the negative predictive value (NPV) was 92.7%. Four women had negative tests on day 5 (β-hCG < 4.0 IU/L), and were positive in the β-hCG day 10 (false negative outcomes), while the remaining four women had a positive test on day 5 (β-hCG ≥ 4.0 IU/L) that was not confirmed in the β-hCG test on day 10 (false positive outcome).

In one patient with positive biochemical pregnancy (β-hCG concentration on day 10 ≥ 25.0 IU/L) the gestational sac was not seen upon the ultrasound. That patient also had β-hCG concentration on day 5 ≥ 4.0 IU/L. The clinical pregnancy rate, defined by the visualization of a gestational sac and the presence of a heartbeat, was 48.3% and the live birth rate was 37.9%. The multiple pregnancy rate was 20.0%.

## DISCUSSION

Generally, a single quantitative measurement of β-hCG levels, 10-12 days after embryo transfer in IVF cycles is an excellent method to diagnose pregnancy (Poikkeus *et al*., 2002). Several studies have evaluated β-hCG concentration as a predictor of pregnancy outcomes after IVF. However, the β-hCG concentrations are generally measured at least 10 days (Kathiresan *et al*., 2011), 11 or 12 days (Qasim *et al*., 1996), 12 days (Poikkeus *et al*., 2002; Lawler *et al*., 2011; Asvold *et al*., 2014; Dypvik *et al*., 2018; Tanbo *et al*., 2018), 13 days (Porat *et al*., 2007; Reljič *et al.*, 2013), 12 and 13 days (Carmona *et al*., 2003), 12 and 14 days (Sung *et al*., 2016), 12 and 24 days (Stone *et al*., 2006), 14 and 21 days (Sugantha *et al*., 2000; Chi *et al*., 2010), and 15 to 35 days after embryo transfer (Delbaere *et al*., 2008). Our study aimed to evaluate whether the serum β-hCG values on day 5 after embryo transfer can be used to diagnose biochemical pregnancy after frozen-thawed IVF cycles, as well as to determine the appropriate cut-off values. We found that a single measurement of β-hCG concentration on day 5 after embryo transfer ≥ 4.0 IU/L can be used as an early diagnostic marker of biochemical pregnancy, with high sensitivity and specificity.

Although hCG can be detected as early as 8-11 days after ovulation, shortly after implantation, in IVF cycles the hCG is commonly used during ovarian stimulation, as well as to trigger final oocyte maturation. Indeed, exogenous hCG leads to false positive tests if the β-hCG concentration is measured before the clearance. On the other hand, pharmacodynamics studies have shown that the complete clearance of exogenous hCG occurs 8 to 12 days after the last application, and that the β-hCG measured in serum on days 12-13 after embryo transfer is of trophoblastic origin (Liu *et al*., 1988). This rationale has been used to justify the β-hCG measurements only after 10 days of embryo transfer, in order to assure complete clearance of exogenous hCG administered during ovulation induction and/or trigger in fresh transfer cycles.

Our study was careful to evaluate only frozen-thawed embryo transfer cycles, in which the patients underwent hormone replacement with estradiol and progesterone, and then there was no influence of exogenous hCG. Hence, it was possible to determine the β-hCG level as early as 5 days after embryo transfer to predict pregnancy with no influence from ovarian stimulation or trigger drugs. In addition, the high sensitivity of the biochemical tests enabled accurate determination of β-hCG concentrations as low as 1 IU/L, which renders a cut-off of 4 IU/L, as defined in our study.

With regards to defining the accuracy of the β-hCG levels on day 5 to diagnose pregnancy, a short definition of metrics used in this study can be appropriate. Sensitivity and specificity are concerned with the accuracy of a screening test relative to a reference standard, while PPV and NPV indicate the effectiveness of a test for categorizing people by the presence or absence of a target condition (Trevethan, 2017). Despite the outcomes showing that all metrics (sensitivity, specificity, PPV, and NPV) were very similar and all over 90%, the use of PPV and NPV to determine the effectiveness of the use of β-hCG 5 days after frozen embryo transfer to pregnancy diagnosis seems to be more appropriate. We found that the probability that people with a positive screening test indeed have the condition of interest (i.e., identifying true positives; here, the positive biochemical test on day 5) was 93.4% (PPV). In addition, the probability of correctly identifying people who do not have the condition (i.e., identifying true negatives) was 92.7% (PNV), showing the accuracy of the measurement of β-hCG on day 5 to accurately diagnose pregnancy.

A limitation of our study is the possible influence of multiple pregnancies. Twin pregnancies result in higher β-hCG levels, which could be a bias in the analysis as 12 from 60 positive pregnancies (20.0%) were twins. However, we performed a supplementary analysis excluding multiple pregnancies and obtained similar outcomes of β-hCG concentration sensitivity and specificity on day 5 ≥ 4.0 IU/L to be used as early diagnostic of pregnancy (data not shown). However, the mean β-hCG value on day 5 for those 12 patients was 28.1 IU/L, which is much higher than the cutoff established to predict pregnancy in general (4.0 IU/L). This suggests that the β-hCG values on day 5 follow the same pattern of those practiced in the routine of pregnancy diagnosis (β-hCG measured on day 10), when higher values can also indicate multiples pregnancies.

Another necessary consideration relies on the supposed false-positive and false-negative tests. In our study, four women who had positive β-hCG on day 5 were negative for β-hCG on day 10 (3.4%), which should be considered as a false positive outcome. In this case, we can argue that those women had a real positive test on day 5, diagnosing a biochemical pregnancy that did not progress, as there is no other possible origin of β-hCG than the embryo. In contrast, the four women who had negative β-hCG concentrations on day 5 and were positive for β-hCG concentration on day 10 (3.4%), were considered as false negatives. The last situation can be considered a genuine false negative outcome, where the levels of β-hCG were lower than the established cut-off for pregnancy diagnosis.

However, it is important to highlight that this is not a prospective controlled study, the data were obtained retrospectively and represent a real-world IVF practice. Thus, some possible bias should be addressed. The very early biochemical pregnancy test, 5 days after FET, can be influenced by the blood collection time, and the patients were instructed that it should be performed at least 120 hours after embryo transfer. However, the blood was collected in a tertiary laboratory, and the time between embryo transfer and blood collection was not strictly controlled. Moreover, variations in time of implantation can also represent a bias. The embryos were transferred when the blastocyst stage was attained, on days 5 or 6 of development according to routine, which can lead to different implantation times and consequently affect the early levels of maternal β-hCG. Hence, those are possible bias of our study and despite the low incidence of false negative tests (3.4%), those factors can be associated to their occurrence.

Very early pregnancy diagnosis after IVF offers a number of advantages. IVF patients are physically and psychologically demanding, and their anxiety levels vary during the treatment. The period preceding the pregnancy test is one of the periods with a high level of anxiety (Harata *et al*., 2012). We intend to continue this study and evaluate how the very early pregnancy test (β-hCG performed on day 5 after embryo transfer) could affect the patient's anxiety level. Another point is the luteal phase support after frozen-thawed embryo transfer, in which progesterone supplementation is routinely administered until at least the day of the pregnancy test, and prolonged until 8 weeks if pregnancy is achieved (Tomic *et al*., 2019). Once pregnancy can be diagnosed earlier, luteal support with progesterone can be managed accordingly.

It is important to highlight that the findings of our study are reliable for FET, and despite the possible bias inherent to a retrospective study, it represents the clinical practice. Our findings showed the very early diagnosis of pregnancy is feasible and accurate by measuring the β-hCG levels as early as 5 days after FET. On the other hand, once the complete clearance of exogenous hCG occurs 8 to 12 days after the last application (Liu *et al*., 1988), the use of β-hCG levels as early as 5 days after embryo transfer using the parameters stablished here, is not applicable for fresh transfers when hCG is used in the stimulation or trigger protocol. Also, it remains unknown whether the β-hCG levels after fresh or frozen-thawed embryo transfer cycles are similar (Reljič *et al*., 2013; Sung *et al*., 2016), and the generalization of our findings for fresh transfers must be validated.

To the best of our knowledge, this is the first study to analyze the predictive value of serum β-hCG concentrations as early as 5 days after frozen-thawed embryo transfer cycles to pregnancy diagnosis. Our data demonstrate that a cut-off value for β-hCG on day 5 after embryo transfer ≥ 4.0 IU/L is accurate for the diagnosis of biochemical pregnancy, and its use in clinical practice enables earlier management, counselling of patients, and appropriate follow-up.
